# Tetanus a Communicable Disease

**Published:** 1890-01-11

**Authors:** 


### "TETANUS, A COMMUNICABLE DISEASE."

Three or four years ago the suggestion that tetanus was a specific and communicable disease would have been rejected as in the highest degree improbable, if not impossible; but already the bacillus of Nicolaier and Rosenbach has made good its claims to recognition from the fact of its being easily cultivated out of the body, and producing the same phenomena in other animals as in man. The last recorded illustration is afforded by Dr. C. Hägler, a patient of whose suffered from gangrene of one foot after a severe attack of enteric fever, and ultimately died of tetanus. The fluids of the gangrenous part contained the bacillus, and eight animals in whom pure cultivations were injected developed typical tetanus. In fact, there can no longer be any doubt that this is one of what may be called extra-corporeal contagia, the microbes of which are capable of existing and perpetuating themselves outside and independently of the bodies of men or animals, and perhaps are as universally distributed as are the spores of the moulds which appear on organic matter whenever the requisite conditions of temperature and moisture are present. Erysipelas, diphtheria, enteric fever, and some other diseases notoriously contagious, yet appearing at times to arise *de novo*, belong to the same class, and thus is explained the frequent occurrence of tetanus after wounds in which dirt or dust has gained admission, while the vulgar notion that those of certain parts of the hand and foot are specially dangerous arises from the circumstances under which such injuries are commonly incurred.

